# Sirt6 regulates efficiency of mouse somatic reprogramming and maintenance of pluripotency

**DOI:** 10.1186/s13287-018-1109-5

**Published:** 2019-01-10

**Authors:** Peng Xu, Ting-ting Wang, Xiu-zhen Liu, Nan-Yu Wang, Li-hong Sun, Zhu-qin Zhang, Hou-zao Chen, Xiang Lv, Yue Huang, De-Pei Liu

**Affiliations:** 1 State Key Laboratory of Medical Molecular Biology, Institute of Basic Medical Sciences, Chinese Academy of Medical Sciences and Peking Union Medical College, Beijing, 100005 China; 2 Department of Biochemistry and Molecular Biology, Institute of Basic Medical Sciences, Chinese Academy of Medical Sciences and Peking Union Medical College, Beijing, 100005 China; 3 Central Research Laboratory, Peking Union Medical College Hospital, Chinese Academy of Medical Sciences and Peking Union Medical College, Beijing, 100730 China; 4 Center for Experimental Animal Research, Institute of Basic Medical Sciences Institute of Basic Medical Sciences, Chinese Academy of Medical Sciences, School of Basic Medicine Peking Union Medical College, Beijing, 100005 China; 5 Department of Medical Genetics, Institute of Basic Medical Sciences, Chinese Academy of Medical Sciences, School of Basic Medicine Peking Union Medical College, Beijing, 100005 China

**Keywords:** Mouse embryonic fibroblast, Reprogramming, Differentiation, Sirt6

## Abstract

**Background:**

Mouse somatic cells can be reprogrammed into induced pluripotent stem cells (iPSCs) by defined factors known to regulate pluripotency, including Oct4, Sox2, Klf4, and c-Myc. It has been reported that Sirtuin 6 (Sirt6), a member of the sirtuin family of NAD^+^-dependent protein deacetylases, is involved in embryonic stem cell differentiation. However, whether and how Sirt6 influences epigenetic reprogramming remains unknown.

**Methods:**

Mouse embryonic fibroblasts isolated from transgenic Oct4-GFP reporter mice with or without Sirt6 were used for reprogramming by Yamanaka factors. Alkaline phosphatase-positive and OCT4-GFP-positive colony were counted to calculate reprogramming efficiency. OP9 feeder cell co-culture system was used to measure the hematopoietic differentiation from mouse ES and iPS cells. RNA sequencing was measured to identify the differential expressed genes due to loss of Sirt6 in somatic and pluripotent cells.

**Results:**

In this study, we provide evidence that Sirt6 is involved in mouse somatic reprogramming. We found that loss of function of Sirt6 could significantly decrease reprogramming efficiency. Furthermore, we showed that Sirt6-null iPS-like cell line has intrinsically a differentiation defect even though the establishment of normal self-renewal. Particularly, by performing transcriptome analysis, we observed that several pluripotent transcriptional factors increase in knockout cell line, which explains the underlying loss of pluripotency in Sirt6-null iPS-like cell line.

**Conclusions:**

Taken together, we have identified a new regulatory role of Sirt6 in reprogramming and maintenance of pluripotency.

**Electronic supplementary material:**

The online version of this article (10.1186/s13287-018-1109-5) contains supplementary material, which is available to authorized users.

## Background

Differentiated somatic cells can be reprogrammed into a pluripotent-like state through four defined factors known to regulate pluripotency, including Oct4, Sox2, Klf4, and c-Myc (OSKM) [1, 2]. Since the discovery of induced pluripotent stem (iPS) cells, the molecular mechanism underlying the reprogramming process has been an active area of research. Recently, alteration of epigenetic state including DNA methylation and discrete alteration of histone modification have been demonstrated to play a critical role during this process [3]. Manipulation of epigenetic modifiers like Tet or Jmjd family protein can either block or enhance reprogramming [4–7]. However, the exact molecular mechanism underlying reprogramming still remains largely unknown. To further understand the epigenetic regulators for specific lineage differentiation from iPS cell would have great significance for potential regeneration therapy and human disease modeling [8].

Sirtuins are conserved in species ranging from bacteria to humans. In mammals, there are seven members (Sirt1–Sirt7) with discrete subcellular localization and have been reported to connect a widening circle of activities including cellular stress resistance, genomic stability, tumorigenesis, and energy metabolism [9]. Sirt1, the mouse homologue of yeast Sir2 deacetylates several non-histone proteins and plays roles in many key functions, including energy metabolism, differentiation, aging, and tumor suppression [10]. Sirt1 was firstly reported to involve in ES cell anti-oxidation and neural progenitor differentiation [11–14]. And later both our group and others reported that Sirt1 can promote the efficiency of reprogramming and maintain characteristics of iPS cell [15–17]. However, whether and how other sirtuins, especially nuclear epigenetic regulator Sirt6, regulate mouse somatic reprogramming still remains exclusive.

Sirt6 was previously reported to regulate many different biological processes like genome stability, glucose metabolism, and tumor suppression [18]. There has been one previous report showing that overexpression of Sirt6 in aged human dermal fibroblasts could improve iPS generation via regulation of miR-766 transcription [19]. During the preparation of our manuscript, another group reported that Sirt6 knockout ES cells skewed towards neuroectoderm differentiation [20]. But the exact role of Sirt6 in mouse somatic reprogramming and iPS cell differentiation remains unrevealed.

In this study, we sought to determine the role of Sirt6 in mouse somatic reprogramming. We found that Sirt6 is highly expressed in pluripotent stem cells and also it regulates the efficiency of somatic reprogramming. Meanwhile, Sirt6-null mouse embryonic fibroblast (MEF)-generated iPS-like cells has intrinsic differentiation defect. Mechanically, we observed that Sirt6-null iPS-like cells failed to maintain expression of several pluripotent transcriptional factors by whole genome RNA sequencing. Therefore, our results indicate that Sirt6 plays a pivotal role in reprogramming and iPS cell fate determination.

## Methods

### Cell culture

mES cell line JM8 (kindly from Yue Huang Lab) was maintained on irradiated feeder layers in knockout Dulbecco’s modified Eagle’s medium (DMEM) supplemented with 15% knockout serum replacement (KSR), glutamine, non-essential amino acids, 100 μM 2-ME, and recombinant leukemia inhibitory factor (LIF). The dishes were coated with 0.1% gelatin [*v*/*v*], and a layer of MMC-treated MEF cells was grown on top as feeder cells. The cells were passaged as needed, and the growth medium was changed daily. Upon harvesting the JM8 cells, the feeder cells were removed by incubation at 37 °C on gelatin-coated dishes for 1.5 h. All MEFs, Plat-E (retroviral packaging cell line, ordered from Cell Biolab, Inc; RV-101), and 293A cells (initially from ATCC, CRL-1573; maintained culture in the lab) were maintained in DMEM containing 10% fetal bovine serum (FBS).

For embryoid body (EB) differentiation, the JM8 cells were suspended in DMEM basic medium (Invitrogen) supplemented with 10% FBS (Invitrogen) and cultured on ultra-low attachment cell culture plate (Corning). For all-trans retinoid acid (ATRA)-induced differentiation, JM8 cells were maintained in ESC culture medium without feeder cells and induced by withdrawal of LIF and the addition of 5 M ATRA (Sigma).

### Gene knockdown and overexpression

For Oct4 knockdown, two siRNAs (Invitrogen) targeting Oct4 and one control siRNA (Invitrogen, 4,404,021) were synthesized. Then, 5 × 10^5^ JM8 cells were transfected with 125 or 100 μM of each siRNA with 5 μl Lipofectamine2000 and cultured on MEF feeder cells for 2 days before harvesting. The siRNA sequences are as follows: SiOct4–1: CGGAAGAGAAAGCGAACTA; SiOct4–2: CGGAAGAGAAAGCGAACTA.

For Sirt6 overexpression, pMXs-based retroviral vectors (Addgene, Yamanaka) encoding the mouse cDNAs of Sirt6 were transfected into PlatE cells using Lipo2000 transfection reagent. Viral supernatant fractions were collected 48 h after transfection and then filtered through a 0.45-μm cellulose acetate filter.

### Animal and MEF derivation

OG2 (Oct4–IRES–GFP/Rosa26-M2rtTA) transgenic mice ubiquitously expressing the enhanced green fluorescent protein (eGFP) under the control of the Oct4 promoter were obtained from Gao SR (University of Tongji, Shanghai, China). Sirt6-null mouse embryonic fibroblasts (MEFs) were derived from Sirt6-null mice (Chuxia Deng’s Lab). All MEFs were derived from 13.5-day mouse embryos and used for induced pluripotent stem cell (iPSC) generation before passage 4.

### Retroviral infection and iPSC generation

**Fig. 1:**
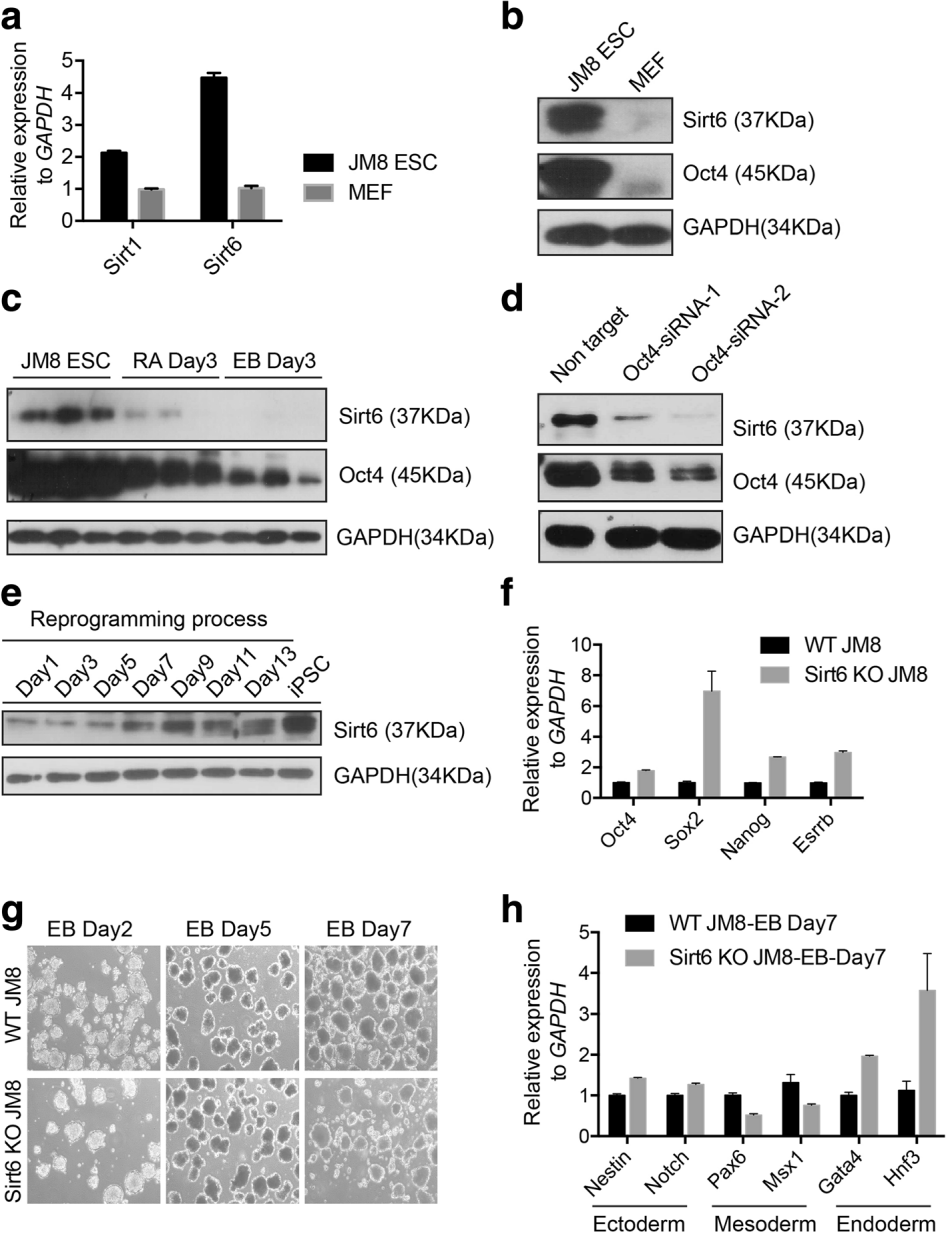
Sirt6 is highly expressed in pluripotent cells relative to differentiated somatic cells. **a** Relative mRNA expression level of Sirt1and Sirt6 in JM8 ESC and MEFs. Data were normalized to the expression levels in MEFs; GAPDH was regarded as internal control gene. Data represent means ± SD of three independent experiments. **b** Western blotting analysis of Sirt6 showed that Sirt6 protein level was much higher in ESCs than in MEFs. **c** Western blotting analysis showed that protein expression of Sirt6 was significantly decreased at day 3 in RA-induced ES differentiation or EB differentiation system. **d** Western blotting analysis of Sirt6 showed that Sirt6 protein level was downregulated in ESCs after RNAi of Oct4. Two siRNA of different sequences (SiOCT4-1 and SiOCT4-2) efficiently knockdown Oct4 expression at protein level. GAPDH was used as the internal control. **e** Western blotting analysis of Sirt6 was shown in different time points (day 1/3/5/7/9/11/13) after MEFs were induced by OSKM factors and also final reprogrammed iPS cell lines. **f** Pluripotency genes (Oct4, Sox2, Nanog, Esrrb) in Sirt6 knockout (KO) ES cells (Additional file 2: Figure S2B and C) were analyzed compared to wild-type iPSCs by qRT-PCR. Data were normalized to the expression levels in wild-type control. Data represented as mean ± SD from three independent assays. **g** EB formation of different time points in Sirt6 KO ES cells compared to wild-type control. **h** Three germ layer marker genes (Nestin and Notch for ectoderm; Pax6 and Msx1 for mesoderm; Gata4 and Hnf3 for endoderm) in Sirt6 KO ES cells were analyzed compared to wild-type control by qRT-PCR during EB formation at day 7. Data represented as mean ± SD from three independent assays

**Fig. 2:**
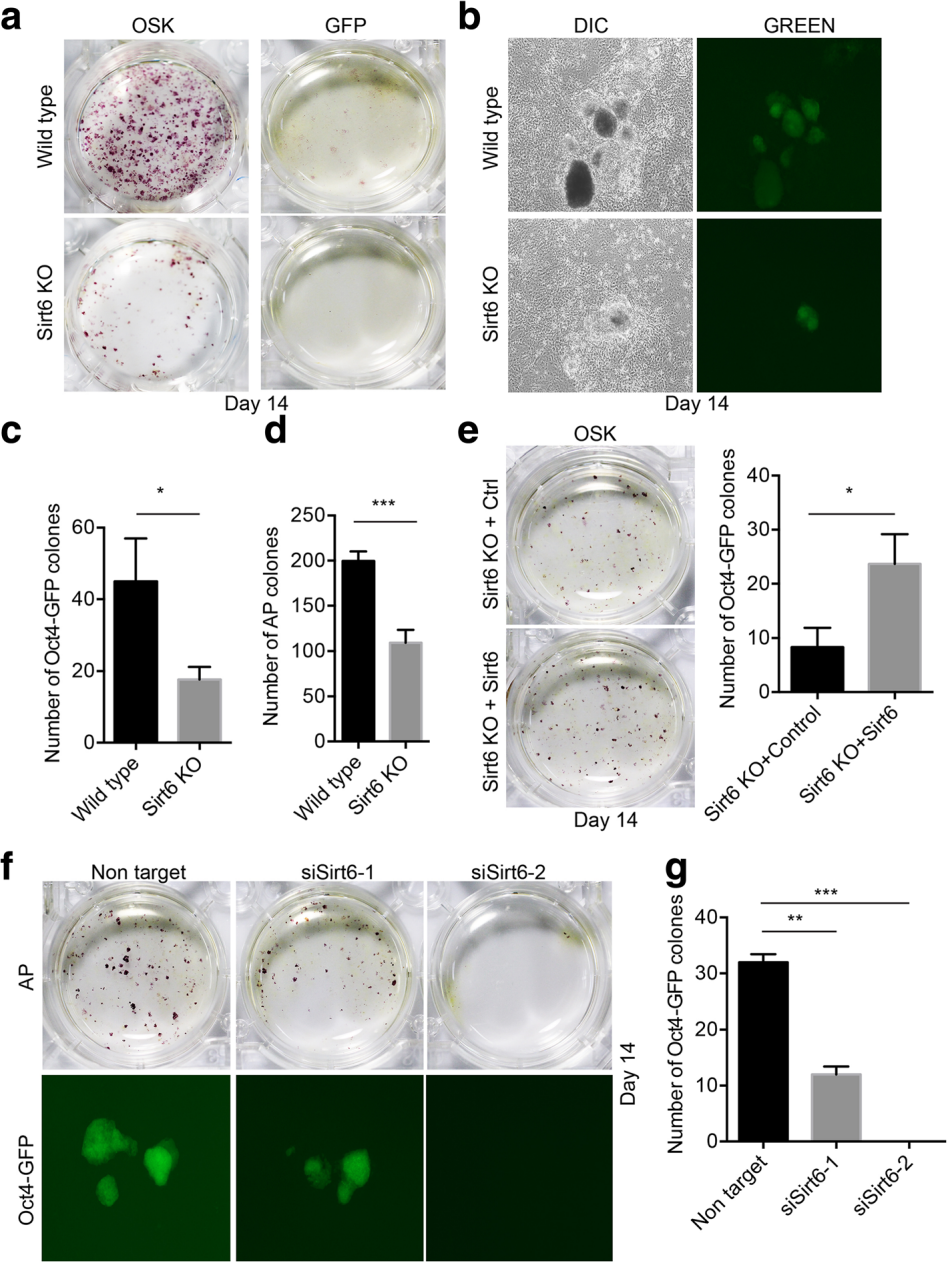
Sirt6 is required for OSK-induced MEF reprogramming. **a** Alkaline phosphatase staining showed reduced MEF reprogramming efficiency in Sirt6-null MEFs. **b** Oct4-GFP-positive clones were shown in both wild-type and Sirt6-null OG2 MEFs after fully reprogramming. **c** Quantification of Oct4-GFP-positive clones showed reduced MEF reprogramming efficiency in Sirt6-null OG2 MEFs. Sirt6-null OG2 MEFs were reprogrammed by introducing OSK, and the number of fully reprogrammed GFP-positive clones was counted. Data represented mean ± SD from three independent assays (*, *p* ≤ 0.05). **d** Quantification of AP-positive clones showed reduced tail-tip fibroblast (TTF) reprogramming efficiency in Sirt6-null TTFs. Sirt6-null TTFs were reprogrammed by introducing OSK. Data represented mean ± SD from three independent assays (***, *p* ≤ 0.001). **e** Oct4-GFP-positive clone counting showed a ~ 2-fold increase in Sirt6-null OG2 MEF reprogramming efficiency upon overexpression of Sirt6. The reprogramming efficiencies were detected using OSK reprogramming factors. The data were shown as mean values ± SD from three independent experiments. (*, *p* ≤ 0.05). **f** Downregulation of Sirt6 decreased the generation of iPSCs. Alkaline phosphatase staining of pre-iPSC clones in wild-type OG2 MEFs transduced with three factors OSK in two siRNA of Sirt6 and in non-target group. **g** The number of GFP-positive clones was counted in wild-type OG2 MEFs transduced with three factors OSK in two siRNA of Sirt6 and in the non-target group. All data were shown as mean values ± SD from three independent experiments. (**, *p* ≤ 0.01; ***, *p* ≤ 0.001)

The pMXs-based retroviral vectors (Addgene, Yamanaka) encoding the mouse cDNAs of Oct4, Klf4, c-Myc, and Sox2 were transfected into PlatE cells using Lipo2000 transfection reagent. Viral supernatant fractions were collected 48 h after transfection and then filtered through a 0.45-μm cellulose acetate filter. For generation of iPSCs, MEFs were infected either with equal amounts of Oct4, Sox2, Klf4, and c-Myc (OSKM, in Fig. 1) or Oct4, Sox2, and Klf4 (OSK, in Fig. 2) viral supernatant fractions in the presence of 8 μg/ml polybrene. Two days after infection, the medium was replaced with fresh ES medium containing knockout serum replacement (KSR) medium, which included LIF. Fourteen days after infection, the iPSC colonies were mechanically isolated and further cultured for identification.

### RNA isolation, real-time reverse-transcriptase polymerase chain reaction analysis, and RNA sequencing

The cells were lysed with TRIzol reagent (Invitrogen), and total RNA was purified according to the manufacturer’s instructions. One microgram of DNaseI-treated RNA was used for cDNA synthesis using a Superscript II transcription kit (Takara). The quantitative real-time PCR (qPCR) reaction was performed in an ABI7500 Fast Real-Time PCR System as per the manufacturer’s instructions.

Total RNA samples were sequenced by Majorbio technology Inc. using Illumina HiSeq2000, with 10 M reads per sample with an average length of > 49 bp. Significance analysis of RNA-seq data was used to identify those genes significantly up- or downregulated by treatments using unlogged data, with a false discovery rate (FDR) less than 0.05. Fold-change was computed with average transcript levels compared to control values, which was in turn log2-transformed and computed for Spearman correlation coefficients between samples. Differential gene expression was calculated by SAM analysis.

### Alkaline phosphatase, immunofluorescence staining, and immunoblot

Alkaline phosphatase (AP) staining was performed with the Alkaline Phosphatase Detection Kit (Millipore), following the supplier’s instructions. Immunofluorescence (IF) staining was performed using primary antibodies (all at 1:200 dilutions) to detect Oct4 (Abcam), Sox2 (Abcam), and Nanog (Abcam). Nuclei were counterstained with 2-(4-amidinophenyl)-6-indolecarbamidine dihydrochloride (DAPI).

Western blot analysis was performed using whole-cell extracts prepared by disrupting the cells in NP40 lysis buffer. Antibodies against GAPDH (Santa Cruz) were used to assess the purity of the respective fractions. The protein bands were visualized using an enhanced chemiluminescence reagent after hybridization with a horseradish peroxidase (HRP)-conjugated secondary antibody. All the Western blot results were quantified by using ImageJ. The antibodies used in this study included anti-Sox2 (Santa Cruz), anti-Sirt1 (Santa Cruz), anti-Myc (Santa Cruz), anti-Oct4 (Santa Cruz), anti-mSSEA1 (Sigma), and anti-Nanog (Cayman).

### Teratoma and chimera formation assay

Cells from a confluent 10-cm plate were harvested by digestion with 2 mg/ml dispase, resuspended in Matrigel, and injected subcutaneously into immunodeficient mice. Eight weeks after injection, teratomas were dissected, fixed in 4% paraformaldehyde, and processed for hematoxylin/eosin (HE) staining.

To produce chimeric mice, approximately 10 iPS cells were microinjected into the ICR (Institute of Cancer Research) eight-cell embryos using piezo-actuated microinjection pipette. After culturing for 1 day, the embryos were transplanted into the uterus of pseudopregnant mice [21].

### Hematopoietic differentiation from mouse ES and iPS cells

For mES and iPS cell differentiation, OP9 cells were plated onto six-well plates in α-MEM containing 20% FBS (HyClone) and 2 mM L-glutamax. Mouse ES cell line or iPS-like cell line was cultured immediately in mES pre-differentiation medium (kDMEM medium contained 15% ES-cult FBS, 2000 U/mL LIF, 2 mM L-glutamax, 0.1 mM non-essential amino acid, 0.1 mM MTG) for 2–3 days after being passaged; then, these cells were digested into single cells by 0.05% Trypsin to generate EBs, and the EBs were suspension cultured in EB differentiation medium (IMDM contained 15% ES-cult FBS, 2000 U/mL LIF, 2 mM L-glutamax, 0.1 mM non-essential amino acid, 0.15 mM MTG, 40 ng/ml mSCF) for 2–3 days; then, the EBs were digested by 0.05% Trypsin into single cells and be added to confluent OP9 cultures and be co-cultured with OP9 cells for 6 days in differentiation medium (α-MEM contained 20% ES-cult FBS,2 mM L-glutamax, 0.15 mM MTG, 40 ng/ml mSCF, 10 ng/ml mIL-3, 10 ng/ml mIL-6, 2u/ml rhEPO). Cells were harvested, and single-cell suspension was prepared for real-time PCR and flow-cytometric assays.

### Flow cytometry analysis

To analyze the efficiency of hematopoietic differentiation, mouse embryonic stem cell (mESC)/OP9 and iPSC/OP9 co-culture were collected and washed with FACS buffer. Cells were stained with c-Kit-PE-Cy7, then they were washed and resuspended in FACS buffer. Gating was done with matched isotype control monoclonal antibodies. All analyses were performed on a BD Accuri C6 Flow analyzer.

### Reporter gene assays

Cells were harvested 36 h after transfection and analyzed for firefly luciferase activity. Transfection efficiency was normalized using a Renilla plasmid as an internal control.

### Statistical analysis

The data are expressed as the mean ± SD of at least three independent samples. Statistical comparisons between groups were performed with a two-tailed Student’s *t* test; *, *p* ≤ 0.05; **, *p* ≤ 0.01; and ***, *p* ≤ 0.001 were considered significant.

## Results

### Sirt6 is highly expressed in pluripotent cells relative to differentiated somatic cells

From previous reports, Sirt1, one main member of sirtuins, was highly expressed in mouse ES cells relative to differentiated mouse embryonic fibroblasts [12]. We hypothesized that another nuclear member Sirt6 may also involve in the stem cell regulation. Real-time PCR was performed to detect Sirt6 in JM8 ES and MEF cells. The result showed that mRNA level of Sirt6 was higher expressed by nearly fourfold in ES cell compared to MEF (Fig. 1a). Consistent with the mRNA level, Sirt6 protein expression level was also higher in ES cells than that in MEF cells (Fig. 1b).

To further compare the expression specificity of Sirt6 in mES cells, we performed RA-induced differentiation and also embryoid body (EB) formation from JM8 ES cells. Similarly, we showed that the expression of Sirt6 largely decreased during the RA-induced and EB differentiation at day 3 (Fig. 1c, Additional file 1: Figure S1A).

Given that Oct4 is a core pluripotent transcription factor in maintaining self-renewal of ES cell and downregulation of Oct4 could cause ES cell differentiation [22], we further used two different siRNAs to knockdown Oct4 in ES cells. The results showed that Sirt6 expression was significantly decreased in response to downregulation of Oct4 (Fig. 1d). We also performed OSKM-induced reprogramming to detect the expression profile of Sirt6 in this process. Temporal expression of Sirt6 protein was gradually increased from day 1 to day 13 during OSKM-induced reprogramming in MEFs (Fig. 1e). To further investigate the role of Sirt6 in ES cells, we generated a Sirt6 knockout (KO) ES cell line by CRISPR-Cas9 (Additional file 1: Figure S1B and C). Importantly, core pluripotency genes including Oct4, Sox2, Nanog, and Esrrb were increased in Sirt6 KO ES cell lines (Fig. 1f). Although Sirt6 KO ES cell could form EBs with typical structure (Fig. 1g), it was shown to have increased expression of ectoderm marker genes (Nestin and Notch) while decreased expression of mesoderm marker genes (Pax6 and Msx1) relative to control (Fig. 1h).

All these results suggest that highly expressed Sirt6 in pluripotent cells might play a role in pluripotency establishment or maintenance.

### Sirt6 is required for OSK-induced reprogramming

To explore whether Sirt6 is required for iPSC generation, we carried out reprogramming of wild-type and Sirt6-null MEFs. To confirm the effect of Sirt6 during reprogramming by scoring fully reprogrammed iPSCs on the basis of Oct4 expression in MEFs carrying an Oct4-GFP knock-in reporter allele, we generated OG2 knock-in and Sirt6-null hybrid homozygous (Sirt6-null OG2) mice (Additional file 2: Figure S2A). Sirt6 expression was validated by real-time PCR, and the results confirmed the loss of expression of Sirt6 (Additional file 2: Figure S2B). Then, we reprogrammed Sirt6-null OG2 MEFs by reprogramming factors Oct4, Sox2, and Klf4.

Firstly, analysis of colony morphology and the number of AP-positive colonies revealed a significant decrease in the reprogramming efficiency of Sirt6-null MEFs relative to wild-type cells (Fig. 2a). Consistently, more than a twofold decrease in reprogramming efficiency was observed in Sirt6-null OG2 MEFs after OSK transduction by counting Oct4-GFP-positive clones (Fig. 2b, c). We also recapitulate this finding in adult mouse tail-tip fibroblast-derived reprogramming (Fig. 2d and Additional file 3: Figure S3C and D). Since the reprogramming of Sirt6-null MEFs was significantly less efficient than that of wild-type cells, we investigated whether overexpression of Sirt6 could restore the reprogramming efficiency in Sirt6-null MEFs. We constructed the pMX-Sirt6 and validated its expression in Sirt6-null MEFs (Additional file 2: Figure S2C). We carried out OSK-mediated reprogramming of OG2/Sirt6-null MEFs by adding Sirt6 or corresponding control. Overexpression of Sirt6 resulted in significant increase of AP-positive clones and also Oct4-GFP-positive clones in the Sirt6 KO MEFs (Fig. 2e).

To further demonstrate whether the positive effect of Sirt6 is a dose-dependent expression effect, we further designed two siRNA sequences which are targeting different regions of Sirt6. The expression levels of siRNA were validated in wild-type MEF firstly, and the results showed that siRNA-1 decreased the Sirt6 expression level by 50% while siRNA-2 decreased by 80% (Additional file 2: Figure S2D). And we further performed reprogramming by using these transient Sirt6 suppression MEFs. To measure AP-positive clone and Oct4-efficiency, we observed that siRNA-1 can decrease reprogramming efficiency by twofold while siRNA-2 almost blocks the reprogramming (Fig. 2f, g). Taken together, these results indicate that Sirt6 plays an important role in OSK-induced iPS cell generation.

Having demonstrated that Sirt6 played a role in somatic reprogramming, next we tried to find out the underlying mechanism. Reprogramming associates with multiple biological processes, including cell apoptosis and cell proliferation. By measuring cell apoptosis in early stage of reprogramming, we found obviously increase of cell death in Sirt6 knockdown group (Additional file 2: Figure S2E). However, interfering with Sirt6 expression by two Sirt6 RNAi retroviral vectors did not affect cell proliferation in OSK (Additional file 2: Figure S2F). These results suggest that Sirt6 might regulate cell reprogramming by protecting cells from apoptosis in early stage.

### Establishment and characterization of Sirt6-null iPS-like cell line

**Fig. 3:**
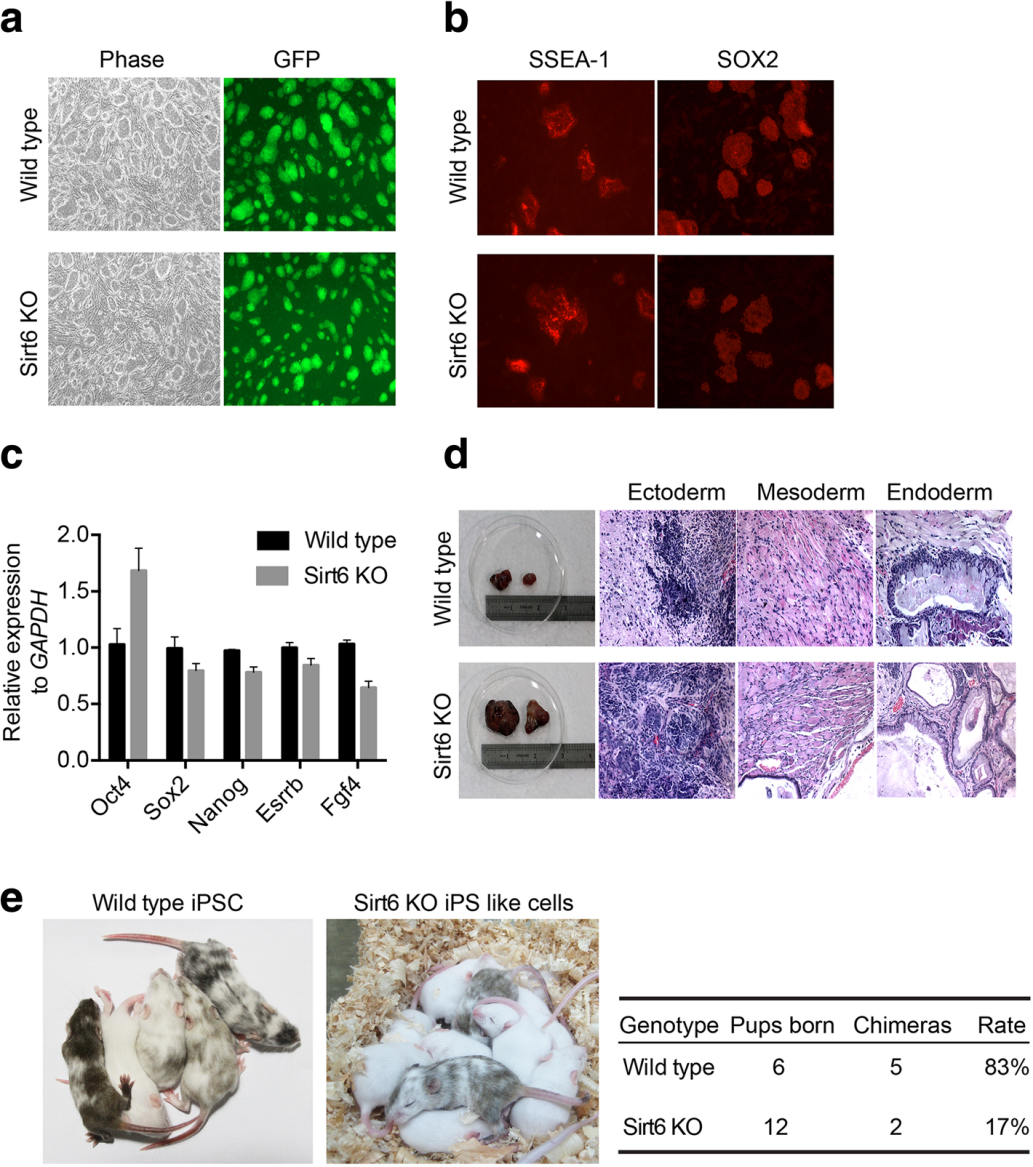
Establishment and characterization of Sirt6-null iPS-like cell line. **a** iPSC clones derived from wild-type and Sirt6 knockout Oct4-GFP MEF cells. iPS clones were picked and proliferated; GFP+ clones were considered as fully reprogrammed. **b** Immunofluorescence analysis showed the expression of SSEA-1 and Sox2 in Sirt6-null iPS-like cells were analyzed compared to wild-type iPSCs (red, antibody staining; blue, Hochest staining). **c** Pluripotency genes (Oct4, Sox2, Nanog, Esrrb, and Fgf4) in Sirt6-null iPS-like cells were analyzed compared to wild-type iPSCs by qRT-PCR. Data were normalized to the expression levels in wild-type control. Data represented as mean ± SD from three independent assays. **d** Teratoma formation in Sirt6-null iPS-like cells compared to wild-type iPSCs. H&E staining was performed after 4 weeks, and all of the above cells formed some typical three germ layers (bars indicated 100 μm). **e** Chimera formation in Sirt6-null iPS-like cells compared to wild-type iPSCs. Chimera as indicated by the agouti coat color; the rate of chimera formation was shown as table in the right panel

Although Sirt6 knockout MEFs have lower reprogramming efficiency by counting the Oct4-GFP-positive clone formation, we can still get small number of Oct4-GFP-positive clones. We picked up several clones with typical ES cell morphology from both wild-type and Sirt6-null MEF, and we defined Sirt6-null clones as “Sirt6-null iPS-like cell line.” And we found these clones can keep more than 10 passages with highly GFP-positive signal, which indicates normal Oct4 activity (Fig. 3a). Pluripotency determination experiments revealed alkaline phosphatase activity in these cells (Additional file 3: Figure S3A). Immunofluorescence also showed the normal expression of SSEA-1 and SOX2 (Fig. 3b). Real-time PCR results demonstrated the normal expression of the ESC marker genes Nanog, Sox2, Esrrb, and Fgf4 and even higher expression of core pluripotency marker Oct4 (Fig. 3c).

To further identify the differentiation ability of Sirt6-null iPS-like cell line, we performed the teratoma formation experiment. We found that Sirt6-null iPS-like cell line was able to form teratoma with larger size compared to wild-type iPS cell line with some of typical three germ layers (Fig. 3d). To convince the quality of generated Sirt6-null iPS-like cell, chimera formation experiment was performed and the results showed that Sirt6-null iPS-like cell had the ability to form chimera mice but with less efficiency (Fig. 3e).

### Sirt6-null iPS-like cell line has differentiation defect

**Fig. 4:**
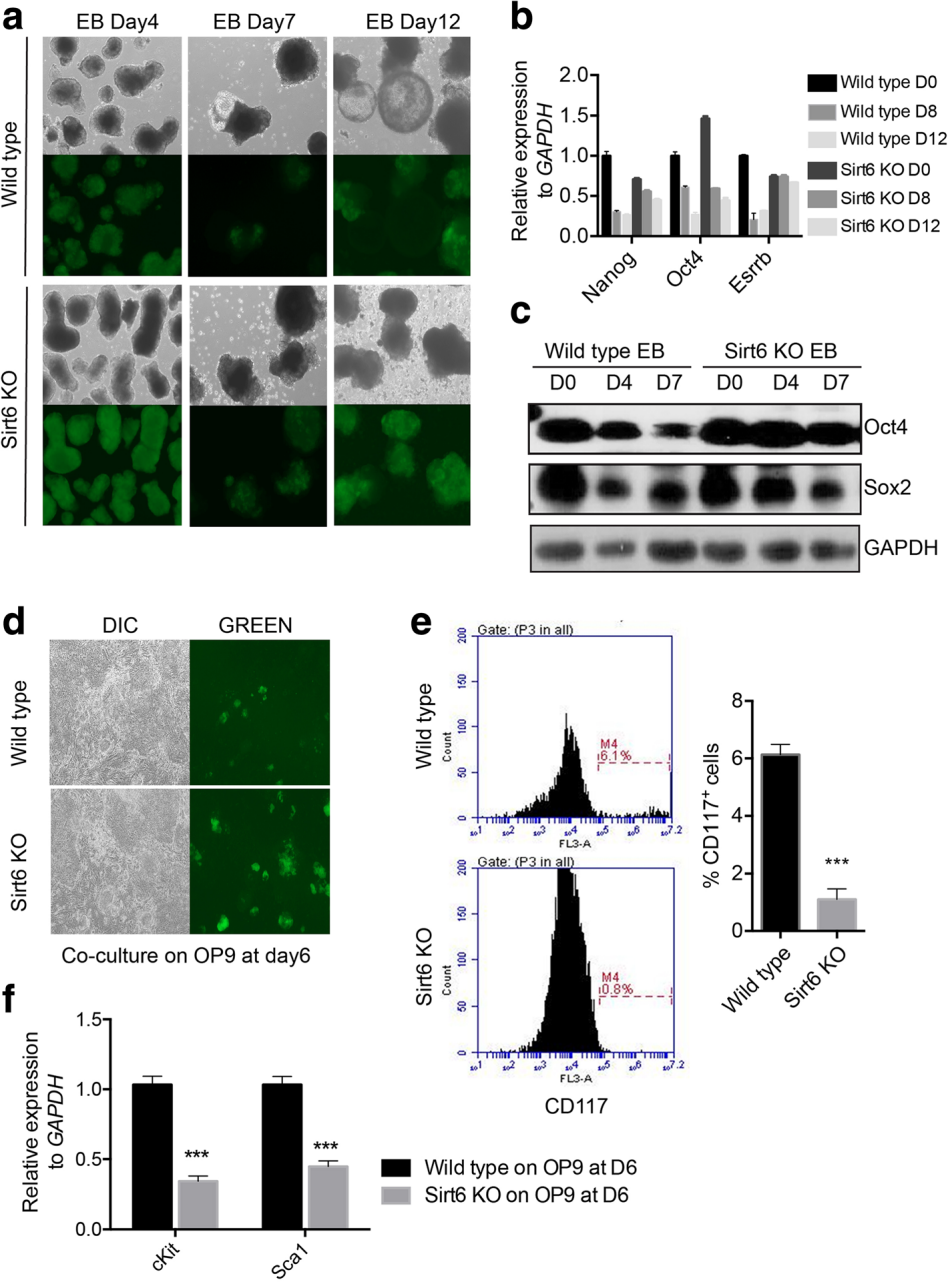
Sirt6-null iPS-like cell line has differentiation defect. **a** EB formation of different time points in Sirt6 knockout iPS-like cells compared to wild-type control. **b** Pluripotency genes (Nanog, Oct4, Esrrb) in Sirt6-null iPS-like cells were analyzed compared to wild-type iPSCs by qRT-PCR during EB formation. Data were normalized to the expression levels in ESCs. Data represented as mean ± SD from three independent assays. **c** Western blot analysis showed the expression of Oct4 and Sox2 in Sirt6-null iPS-like cells compared to wild-type iPSCs in EB differentiation. **d** GFP-positive cell retained more percentage in Sirt6-null iPS-like cells on OP9 co-cultured hematopoietic differentiation system, 50×. **e** c-kit + cells percentage by FACS in day9 on OP9 co-cultured hematopoietic differentiation from wild-type iPS cells and Sirt6-null iPS-like cells. **f** c-Kit and Sca1 gene expression levels were analyzed by qRT-PCR in wild-type iPS cells and Sirt6-null iPS-like cells induced hematopoietic progenitors at day 9 on OP9 co-cultured hematopoietic differentiation system. Data were normalized to the expression levels in wild-type control. Data represented as mean ± SD from three independent assays

To further measure the differentiation ability of Sirt6-null iPS-like cell line, we performed embryoid body (EB) from both wild-type iPS cell line and Sirt6-null iPS-like cell line. We could not observe a typical vacuolar structure in Sirt6-null iPS-like cell line compared to wild-type iPS cells; meanwhile, we found a much higher Oct4-GFP-positive signal in Sirt6-null iPS-like cell-generated EBs (Fig. 4a). Further, we found Oct4, Sox2, and Nanog failed to downregulate during EB formation by real-time PCR and Western blot (Fig. 4b, c). These results suggest that Sirt6-null iPS-like cell line loses the ability to form normal EBs.

Since Sirt6-null ES line showed mesodermal marker genes block (Fig. 1h) during EB differentiation and also from a recent report [20], we further established hematopoietic differentiation system to measure both Sirt6-null iPS-like cell line and wild-type control. Firstly, we used mouse ES cell line as a control, which can show typical hematopoietic differentiation efficiently on OP9 stroma cells (Additional file 4: Figure S4). Then, we performed this assay in both wild-type iPS cell and Sirt6-null iPS-like cell line. We found that during the differentiation, Sirt6-null iPS-like cell still remained more Oct4-GFP-positive cells compared to wild-type iPS cell line (Fig. 4d). And by using CD117 as a marker for hematopoietic lineage marker, we found significant less CD117+ cells in Sirt6-null iPS-like group (Fig. 4e). Consistent with this morphology change, mesodermal markers Nkx2.5 and Mesep1 were both failed to upregulate in Sirt6-null iPS-like cell-derived hematopoietic progenitors (Fig. 4f).

### Transcriptome analysis identifies differential expressed genes in Sirt6-null iPS-like cells and Sirt6-null MEFs

To further figure out the underlying molecular mechanisms of reprogramming and differentiation defects due to loss of Sirt6, we compared the global transcription profiles in two status of cell context: Sirt6-null iPS-like cell line and Sirt6-null MEFs relative to its correspondent control by RNA transcriptome analysis.

**Fig. 5:**
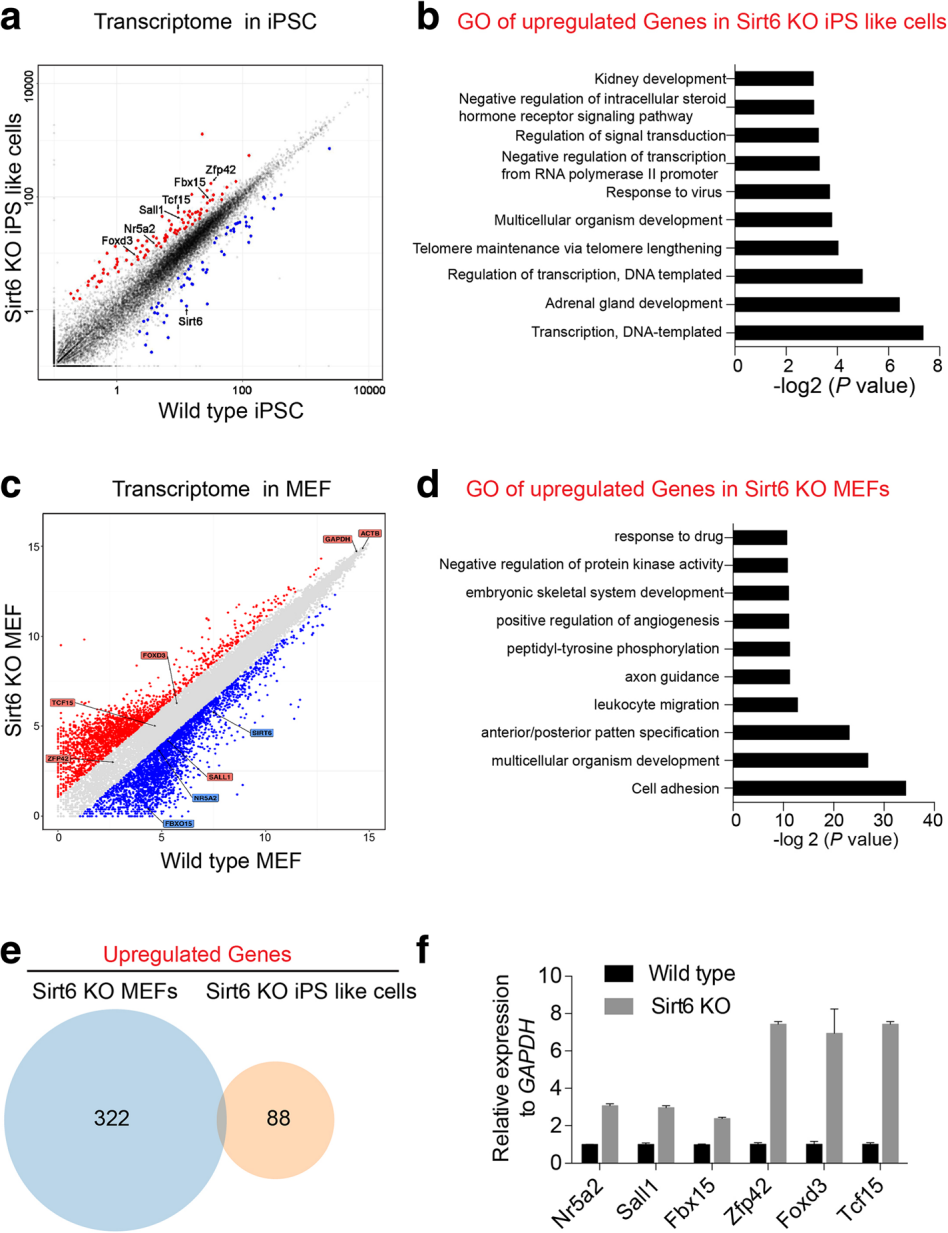
Transcriptome analysis identifies differential expressed genes in Sirt6-null iPS-like cells and Sirt6-null MEFs. **a** Transcriptome analysis in wild-type iPS cell line and Sirt6-null iPS-like cell. Each dot represents an individual gene; differentially expressed genes are depicted according to FPM (fragments per million mapped reads) values. Red dots represent the 63 significantly upregulated genes, and blue dots represent the 144 significantly downregulated genes. Some pluripotency-related genes like Nr5a2, Sall1, Fbx15, Foxd3, and Tcf15 were highlighted and arrowed. **b** Enriched Gene Oncology (GO) terms of the upregulated gene lists in Sirt6-null iPS cell line. **c** Transcriptome analysis in wild-type MEF and Sirt6-null MEF. Each dot represents an individual gene; differentially expressed genes are depicted according to microarray probe signal values. Red dots represent the 325 significantly upregulated genes, and blue dots represent the 457 significantly downregulated genes. Some pluripotency-related genes like Nr5a2, Sall1, Fbx15, Foxd3, and Tcf15 were highlighted and arrowed. **d** Enriched Gene Oncology (GO) terms of the upregulated gene lists in Sirt6-null MEFs. **e** Venn diagrams showing the overlap of upregulated gene lists between Sirt6-null iPS-like cell line and Sirt6-null MEFs. **f** Real-time qPCR analysis of gene expression including Nr5a2, Sall1, Sall3, FbXO15, Zfp42, Foxd3, and Tcf15. Data were normalized to the expression levels in wild-type iPSCs. Data represented as mean ± SD from three independent assays

To firstly investigate the impact of Sirt6 on pluripotent state, we compared genes that were up- or downregulated in Sirt6 null relative to the wild-type iPS cell line and found that 91 genes were upregulated by twofold while 58 genes were downregulated in the Sirt6-null iPS-like cell (Fig. 5a, Additional file 5: Table S1). Among the upregulated gene candidates, we found embryonic self-renewal transcription factors like Nr5a2, Sall1, Fbx15, Zfp42, Foxd3, and Tcf15 were all increased in Sirt6-null iPS-like cell line (Additional file 4: Figure S4C). Further, we analyzed these upregulated genes and downregulated genes for Gene Ontology (GO) annotations. The GO terms significantly enriched in the upregulated gene were specific to DNA binding or regulation of transcription and development-related process (Fig. 5b, Additional file 6: Table S2). In contrast, we further compared genes that were up- or downregulated in Sirt6-null MEFs and observed that 325 genes were upregulated by twofold while 457 genes were downregulated in the Sirt6-null MEFs (Fig. 5c, Additional file 7: Table S3). The GO terms significantly enriched in the upregulated gene were specific to cell adhesion, multicellular organism development, and embryonic skeletal system development (Fig. 5d, Additional file 8: Table S4). Of note, those embryonic self-renewal transcription factors like Nr5a2, Sall1, Fbx15, Foxd3, and Tcf15 were not changed in Sirt6-null MEFs (Fig. 5c). Moreover, genes that are upregulated in Sirt6-null MEFs are largely different from those that upregulated in Sirt6-null iPS-like cells (Fig. 5e). Further RT-qPCR analysis confirmed that pluripotency genes including Nr5a2, Sall1, Fbx15, Zfp42, Foxd3, and Tcf15 were significantly upregulated in the Sirt6-null iPS-like cells (Fig. 5f). Those results suggest that Sirt6 might directly regulate pluripotency gene expression in pluripotent cell state.

### Sirt6 affects Oct4 transcriptional activity on pluripotency genes

As a transcription repressor, Sirt6 could repress genes through deacetylation of H3K56 and H3K9. For instance, Sirt6 functions as H3K9 deacetylase to silence HIF1a target glycolysis genes including Glut1, Pdk4, Pdk1, LDHB, and so on [23]. Recently, Sirt6 was reported to directly regulate levels of H3K56 and H3K9 acetylation at the promoters of Oct4, Sox2, and Nanog during ES-derived EB differentiation [20]. It is likely that Sirt6 also regulate other pluripotency genes.

**Fig. 6:**
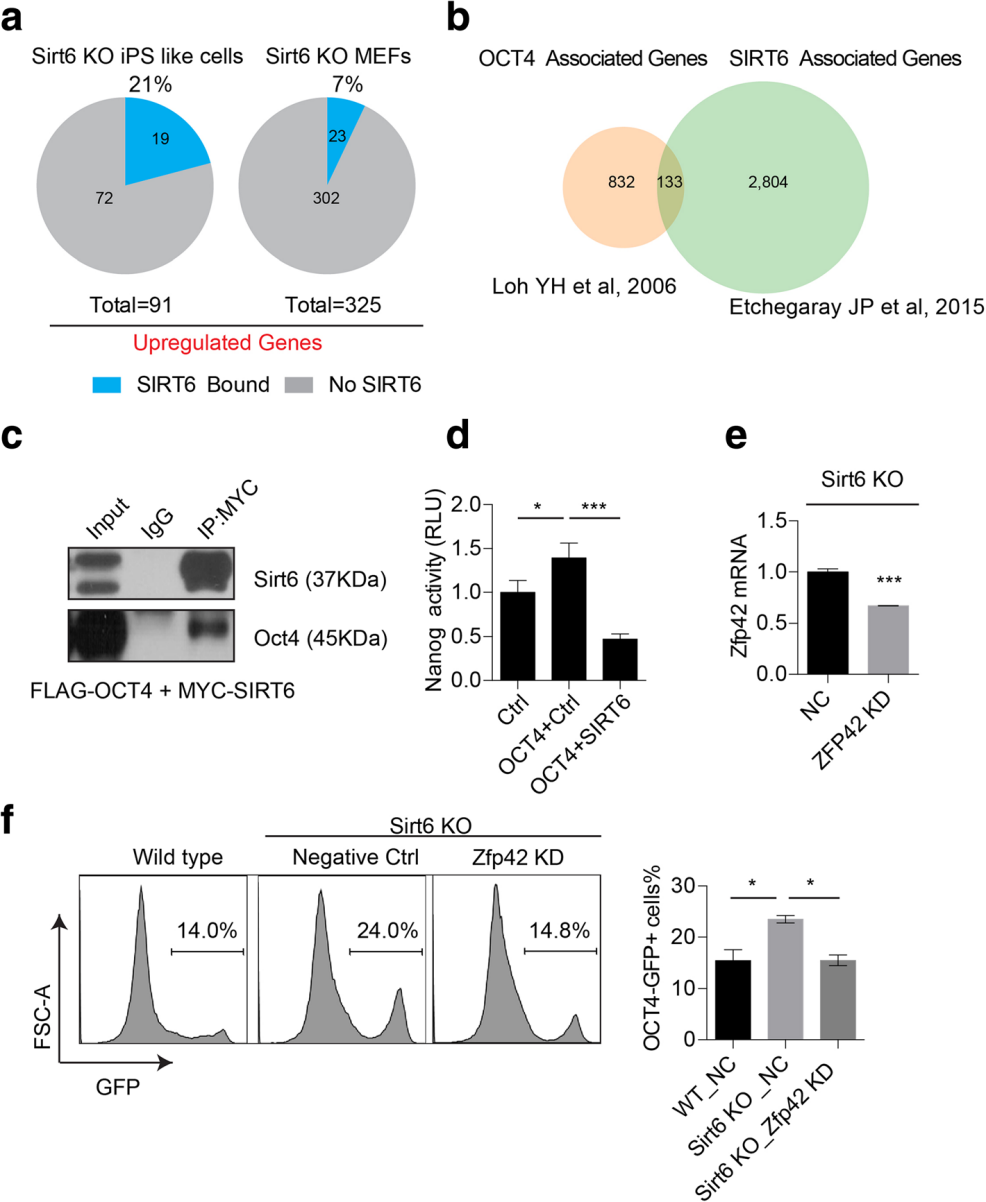
SIRT6 affects OCT4 transcriptional activity on pluripotency genes. **a** SIRT6 binding to upregulated genes in Sirt6-null iPS-like cell line and Sirt6-null MEFs. SIRT6 ChIP-seq published datasets [20] were analyzed by combining with the transcriptome datasets in Fig. 5. **b** Venn diagrams showing the overlapped associated genes of SIRT6 and OCT4 in mouse ES cells [24]. **c** Coimmunoprecipitation results showed that SIRT6 bound with OCT4. FLAG-tagged OCT4 and MYC-tagged SIRT6 were co-transfected in 293T cells and immunoprecipitation was performed by MYC antibody, and Western blotting was performed by indicated antibodies. **d** A luciferase reporter experiment showed the upregulation of Oct4 transcriptional activity on a Nanog promoter reporter and inhibited by co expressed with Sirt6. Oct4 was co-transfected with a Nanog reporter into 293T cells with Sirt6 or with empty vector control; firefly luciferase activity represented Nanog reporter activation level. Data represented as mean ± SD from three independent assays. **e** Real-time PCR for mRNA of Zfp42 in Sirt6-null iPS-like cell line transfected with Zfp42 siRNAs (Zfp42 KD) or negative control (NC) siRNA. **f** Percentage of OCT4-GFP-positive cells after 2 days of RA-induced differentiation of Sirt6-null iPS-like cells transfected with Zfp42 siRNAs (Zfp42 KD) or negative control (NC) compared to wild-type iPSCs. Representative flow cytometry studies are shown on the left, and the results of three replicates are summarized in the graph (*, *p* < 0.05; unpaired *t* test)

By analysis of the public available Sirt6 ChIP-seq datasets [20], we found that 19 out 91 genes (21%) in iPS cells were bound with Sirt6 while only 23 out of 325 genes (7%) were bound with Sirt6 in MEF cells (Fig. 6a). Sirt6-associated genes in mouse embryonic stem cells were found to be partially overlapped with core transcriptional factors Oct4 target genes by integrating Sirt6 ChIP-seq and Oct4 ChIP-seq datasets [24] (Fig. 6b). These results indicate that upregulated genes in Sirt6-null iPS-like cells might be directly regulated by core transcriptional factors coordinated with Sirt6. Moreover, Sirt6 could immunoprecipitate with Oct4 when both tagged version were co-expressed in 293T cells (Fig. 6c), which confirmed that Sirt6 is one of the interactome of Oct4 from previous finding [25]. In addition, we also found Sirt6 could significantly inhibit the transcriptional activity of Oct4 on Nanog promoter-driven luciferase reporter (Fig. 6d). We next test if suppression of those pluripotency genes including Nr5a2, Sall1, Fbx15, Foxd3, Zfp42, and Tcf15 individually could restore the Sirt6-null iPS-like cell differentiation defects to some extent. We found that suppression of Zfp42 in Sirt6-null iPS-like cells could significantly rescue the Oct4 promoter reporter GFP-positive cells comparable to wild-type control cells (Fig. 6e, f) while suppression of other pluripotency genes showed no effect (Additional file 4: Figure S4D). All these results indicate that differentiation potential of Sirt6-null iPS-like cell might be impaired due to loss of the ability to silence a set of self-renewal gene expression.

## Discussion

In this study, we showed that knocking down or knocking out of Sirt6 could significantly decreased MEF reprogramming efficiency, which suggests that Sirt6 is required for efficient somatic reprogramming from mouse embryonic fibroblasts. Our results further showed that Sirt6-null iPS-like cells can be established but this cell line had differentiation defects due to failing to block some pluripotency transcription factor expression based on transcriptome analysis.

Sirt6, as one of nuclear sirtuin members, was firstly reported to locate in the heterochromatin region and could regulate the genome stability in ES cells [26]. In the present study, we found that Sirt6 was highly expressed in mouse JM8 ES cell and was decreased during RA-induced and also EB differentiation (Fig. 1). The maintenance of embryonic pluripotent state is controlled by both transcription factors and the epigenetic modification of the chromatin [27, 28]. Sirt6 was reported as Oct4-interacted protein by mass spectrum [25], and it was validated from this study (Fig. 5f). Further, we also confirmed the differentiation defect from Sirt6 knockout ES cells by CRISPR-Cas9 technology, and the phenotype was consistent with the recent finding [20]. All these evidence suggests a positive role of Sirt6 in ES cell pluripotency regulation.

What is more, Sirt6 was also highly expressed in mouse iPS cells, which is consistent with the finding that high expression of Sirt6 in human iPS cell line compared to human fibroblasts [19]. We also observed that the protein level of Sirt6 was increased after being induced by Oct4, Sox2, Klf4, and c-Myc in mouse embryonic fibroblast reprogramming. One previous genome-wide assay to identify the roadmap of reprogramming also showed that the Sirt6 mRNA level achieved the highest peak at the day 5 [29]. This elevation of Sirt6 in the early stage of reprogramming indicates that Sirt6 might be required for successful reprogramming.

In this work, we found that reprogramming efficiency decreased dramatically in Sirt6-null MEF and by inhibition of Sirt6 in wild-type cells, which was measured by early reprogramming marker alkaline phosphatase (AP) and also late reprogramming marker Oct4 promoter activity. Furthermore, overexpression of Sirt6 could partially rescue the decreased efficiency of Sirt6-null MEF reprogramming. Our study was consistent with the positive role of Sirt6 in promoting aged human cell-derived iPS generation [19] and aged mouse-derived iPS generation [30]. However, one recent work published an increase rather than decrease in iPSC formation during reprogramming from Sirt6 knockout mouse neural progenitor cells from the supplementary evidence [20]. This inconsistency could be explained by at least two reasons. Firstly, a different cell context may require a different epigenetic regulator for reprogramming. In this study, both MEFs and adult tailed-derived fibroblasts from Sirt6 knockout mice showed significantly decreased efficiency of reprogramming, which is different from neural progenitor cell context. Secondly, the reprogramming system is also different from our study. Sirt6 knockout MEFs in our study were derived from two genetic background mice which was OG2 knock-in and Sirt6-null hybrid homozygous (Sirt6-null OG2), so the Oct4 GFP-positive clones were used to analyze the reprogramming efficiency. And further we also used RNAi strategy to measure the transient effect of Sirt6 in reprogramming efficiency. We also reported that Sirt1 enhance reprogramming in our group [17]. Sirt6 has at least two same targets H3K56 and H3K9 from previous study and has similar effect in many biological processes like aging and cancer [23, 31]. Together, we provide evidences to show that Sirt6 plays a positive role in at least mouse embryonic fibroblast reprogramming.

Although we observed that Sirt6-null MEF showed less Oct4-GFP-positive clones after reprogramming for 2 weeks, we could still establish iPS-like cell lines from these clones and we defined this cell line as Sirt6-null iPS-like cell. Based on the lower efficiency of pluripotency, we speculated that Sirt6-null iPS-like cell might not be fully functional iPSCs. We observed that all the clones could expand on feeder cells with ES media for more than 10 passages and also showed large nuclear/cytoplasm ratio, rapid proliferation, and normal Oct4, Sox2, Nanog, and SSEA-1 expression. These results are also consistent with that from a previous study; Sirt6 knockout ES cell line could be generated by typical gene targeting strategy [26]. However, we also observed some different phenotype with some previous work [20, 30]. First, Sirt6-null iPS-like cells tend to have higher expression level of pluripotency marks including Oct4, Nr5a2, Sall1, Fbx15, Zfp42, Foxd3, and Tcf15 in this study (Figs. 3c and 5b), while Sirt6 knockout iPS cells show normal levels of Sox2, Nanog, and Esrrb [30]. This discrepancy might due to the establishment method of reprogramming or the genetic background of MEF cells. Second, in terms of differentiation potential, Sirt6-null iPS-like cell has shown a bigger size of teratoma with some of typical three germ layer tissues (Fig. 3d) and defects in embryonic body structure (Fig. 4a). This is different from the phenotype in Sirt6-null ES cells [20], with a smaller size of teratoma and normal shape of embryonic body structure. Although we could not fully characterize the underlying mechanism or this opposite effect, the different downstream genes regulated by Sirt6 in between iPS or ES might result in a different differentiation effect. Sirt6-null iPS-like cell has also shown that lower efficiency of hematopoietic progenitor cell generation in vitro further indicates the differentiation defect of this cell line although teratoma tissue with normal hematopoiesis tissues in vivo (Fig. 4e, f), which is consistent with a recent study that Sirt6 is required for the hematopoietic stem cell homeostasis [32]. Consistent with the finding that Sirt6 knockout ES cell skewed to neuroectoderm differentiation [20], our study showed that Sirt6 could be another crucial regulator for mesoderm differentiation of iPS cells. Although higher Oct4, Zfp42, Sall3, Nr5a2, and Foxd3 expression were observed in Sirt6 KO iPS-like cells, which might partially explain the differentiation defect, the detailed mechanism underlying this process needs to be further illuminated.

## Conclusions

In summary, our data clearly indicate a positive role of Sirt6 in mouse somatic reprogramming. Furthermore, we observed a differentiation defect in Sirt6-null iPS-like cell line, which suggests a new epigenetic enzyme in regulating pluripotency maintenance.

## Additional files

Additional file 1: **Figure S1.** (A): Sirt6 mRNA level after RA induced JM8A3 ES cells differentiation. All gene mRNA levels were measured by real time PCR; Oct4, Sox2 were represented as positive control, GAPDH was used as reference gene. All data were shown as mean values ± SD from three independent experiments. (*, *p* ≤ 0.05; **, *p* ≤ 0.01; ***, *p* ≤ 0.001). (B): Two CRISPR sgRNAs were designed to knockout region between exon4 and exon5 (highlighted in yellow) (781 bp); PCR primers and fragment used for genotyping was shown on the top. (C): Genotyping of the Sirt6 knockout JM8 ES clones by PCR; One pure knockout clone (D12) was selected. (D): Western blot to validate the expression of Sirt6 in Sirt6 knockout JM8 ES cells. (JPG 542 kb)

Additional file 2: **Figure S2.** (A): Sirt6/OG2 MEF genotype. Two sets of primers were used to genotype Sirt6 and Oct4-GFP allele separately. (B): Validation of Sirt6 mRNA by real-time PCR in Sirt6 wild-type and knockout MEFs. (C): Sirt6 expression measurement by Western blot after overexpression in Sirt6-null MEFs. (D): Validation of Sirt6 expression by real-time qPCR after two siRNA were transient transfection in mES-JM8 cells. (E): Cell apoptosis was measured in wild-type and Sirt6 null during reprogramming (F): Cell proliferation were measured in both wild-type and Sirt6-null MEFs after induced by OSKM at Day0 and Day5. (G): Viable cell numbers in both wild-type and Sirt6-null MEFs were assessed using a colorimetric assay with the light absorbance readout (OD 490 nm) at serial time points. (JPG 1001 kb)

Additional file 3: **Figure S3.** (A): iPSC clones derived from wild-type and Sirt6-null Oct4-GFP MEF cells. IPS clones were picked and proliferated and identified by alkaline phosphatase staining. (B): Sirt6 expression was validated by Western blot in wild-type and Sirt6-null iPSC cells. (C): Tail-tip fibroblasts (TTF) and TTF-derived iPSCs. (D): Alkaline phosphatase staining showed reduced TTF reprogramming efficiency in Sirt6-null TTFs and wild-type TTFs. (JPG 2005 kb)

Additional file 4: **Figure S4.** (A): Three stages of in vitro hematopoietic differentiation from mES cells and iPS cells. Firstly, Mouse ES cell line or iPS-like cell line were cultured immediately in mESC pre-differentiation medium.; then these cells were digested into single cells by 0.05% Trypsin to generate EBs, the EBs were suspension cultured in EB differentiation medium. Finally, the EBs were digested by 0.05% Trypsin into single cells and be added to confluent OP9 cultures, and be co-cultured with OP9 cells for 6 days in differentiation medium. (B): Typical images of different stages of in vitro hematopoietic differentiation from mouse ES cells. (C): FPM showed the expression level of Nr5a2, Sall1, Sall3, Fbx15, Zfp42, Foxd3 and Tcf15 were upregulated in Sirt6-null iPS-like cell line. (D): Percentage of OCT4-GFP positive cells after 2 days of RA induced differentiation of Sirt6-null iPS-like cells transfected with Tcf15, Foxd3, Fbxo15, Sall1, and Nr5a2 siRNAs (Zfp42 KD) or negative control (NC) compared to wild-type iPSCs. Results of 3 replicates are summarized in the graph. (JPG 849 kb)

Additional file 5: **Table S1.** Differential expression genes in Sirt6-null iPS-like cells relative to wild-type controls. (XLSX 30 kb)

Additional file 6: **Table S2.** Gene ontology analysis from downregulated gene sets in Sirt6-null iPS-like cells. (XLSX 41 kb)

Additional file 7: **Table S3.** Differential expression genes in Sirt6-null MEFs relative to wild-type controls. (XLSX 97 kb)

Additional file 8: **Table S4.** Gene ontology analysis from downregulated gene sets in Sirt6-null MEFs. (XLSX 10 kb)

## Abbreviations

AP: Alkaline phosphatase
DMEM: Dulbecco’s modified Eagle’s medium
EB: Embryoid body
FBS: Fetal bovine serum
FDR: False discovery rate
IF: Immunofluorescence
iPSCs: Induced pluripotent stem cells
KSR: Knockout serum replacement
MEF: Mouse embryonic fibroblasts
mESC: Mouse embryonic stem cell
OG2: Oct4-GFP
OSKM: Oct4, Sox2, Klf4, and c-Myc
RA: Retinoid acid

## References

[1] Takahashi K, Yamanaka S (2006). Induction of pluripotent stem cells from mouse embryonic and adult fibroblast cultures by defined factors. Cell.

[2] Takahashi K (2007). Induction of pluripotent stem cells from adult human fibroblasts by defined factors. Cell.

[3] Apostolou E, Hochedlinger K (2013). Chromatin dynamics during cellular reprogramming. Nature.

[4] Chen J (2013). H3K9 methylation is a barrier during somatic cell reprogramming into iPSCs. Nat Genet.

[5] Gao Y (2013). Replacement of Oct4 by Tet1 during iPSC induction reveals an important role of DNA methylation and hydroxymethylation in reprogramming. Cell Stem Cell.

[6] Hu X (2014). Tet and TDG mediate DNA demethylation essential for mesenchymal-to-epithelial transition in somatic cell reprogramming. Cell Stem Cell.

[7] Sridharan R (2013). Proteomic and genomic approaches reveal critical functions of H3K9 methylation and heterochromatin protein-1γ in reprogramming to pluripotency. Nat Cell Biol.

[8] Wu SM, Hochedlinger K (2011). Harnessing the potential of induced pluripotent stem cells for regenerative medicine. Nat Cell Biol.

[9] Finkel T, Deng CX, Mostoslavsky R (2009). Recent progress in the biology and physiology of sirtuins. Nature.

[10] Haigis MC, Sinclair DA (2010). Mammalian sirtuins: biological insights and disease relevance. Ann Rev Pathol.

[11] Calvanese V (2010). Sirtuin 1 regulation of developmental genes during differentiation of stem cells. Proc Natl Acad Sci U S A.

[12] Han MK (2008). SIRT1 regulates apoptosis and Nanog expression in mouse embryonic stem cells by controlling p53 subcellular localization. Cell Stem Cell.

[13] Hisahara S (2008). Histone deacetylase SIRT1 modulates neuronal differentiation by its nuclear translocation. Proc Natl Acad Sci.

[14] Prozorovski T (2008). Sirt1 contributes critically to the redox-dependent fate of neural progenitors. Nat Cell Biol.

[15] De Bonis ML, Ortega S, Blasco MA (2014). SIRT1 is necessary for proficient telomere elongation and genomic stability of induced pluripotent stem cells. Stem Cell Rep.

[16] Lee YL (2012). Sirtuin 1 facilitates generation of induced pluripotent stem cells from mouse embryonic fibroblasts through the miR-34a and p53 pathways. PLoS One.

[17] Mu WL (2015). Sox2 deacetylation by Sirt1 is involved in mouse somatic reprogramming. Stem Cells.

[18] Kugel S, Mostoslavsky R (2014). Chromatin and beyond: the multitasking roles for SIRT6. Trends Biochem Sci.

[19] Sharma A, et al. The role of SIRT6 protein in aging and reprogramming of human induced pluripotent stem cells. J Biol Chem. 2013;288. 10.1074/jbc.M112.405928. 10.1074/jbc.M112.405928 PMC3689986 23653361

[20] Etchegaray JP (2015). The histone deacetylase SIRT6 controls embryonic stem cell fate via TET-mediated production of 5-hydroxymethylcytosine. Nat Cell Biol.

[21] Jiao J (2013). Promoting reprogramming by FGF2 reveals that the extracellular matrix is a barrier for reprogramming fibroblasts to pluripotency. Stem Cells.

[22] Niwa H, Miyazaki JI, Smith AG (2000). Quantitative expression of Oct-3/4 defines differentiation, dedifferentiation or self-renewal of ES cells. Nat Genet.

[23] Zhong L (2010). The histone deacetylase Sirt6 regulates glucose homeostasis via Hif1alpha. Cell.

[24] Loh YH (2006). The Oct4 and Nanog transcription network regulates pluripotency in mouse embryonic stem cells. Nat Genet.

[25] Ding J, Xu H, Faiola F, Ma’Ayan A, Wang J (2012). Oct4 links multiple epigenetic pathways to the pluripotency network. Cell Res.

[26] Mostoslavsky R (2006). Genomic instability and aging-like phenotype in the absence of mammalian SIRT6. Cell.

[27] Boyer LA (2005). Core transcriptional regulatory circuitry in human embryonic stem cells. Cell.

[28] MacArthur BD, Maayan A, Lemischka IR (2009). Systems biology of stem cell fate and cellular reprogramming. Nat Rev Mol Cell Biol.

[29] Polo JM (2012). A molecular roadmap of reprogramming somatic cells into iPS cells. Cell.

[30] Chen W (2017). Sirt6 promotes DNA end joining in iPSCs derived from old mice. Cell Rep.

[31] Kawahara TLA (2009). SIRT6 links histone H3 lysine 9 deacetylation to NF-kappaB-dependent gene expression and organismal life span. Cell.

[32] Wang H (2016). SIRT6 controls hematopoietic stem cell homeostasis through epigenetic regulation of Wnt signaling. Cell Stem Cell.

