# Supplementary figures and images for: Sirt6 regulates efficiency of mouse somatic reprogramming and maintenance of pluripotency
Source: Stem Cell Res Ther. 2019 Jan 10;10:9. doi: 10.1186/s13287-018-1109-5 (PMC6329104; doi:10.1186/s13287-018-1109-5)

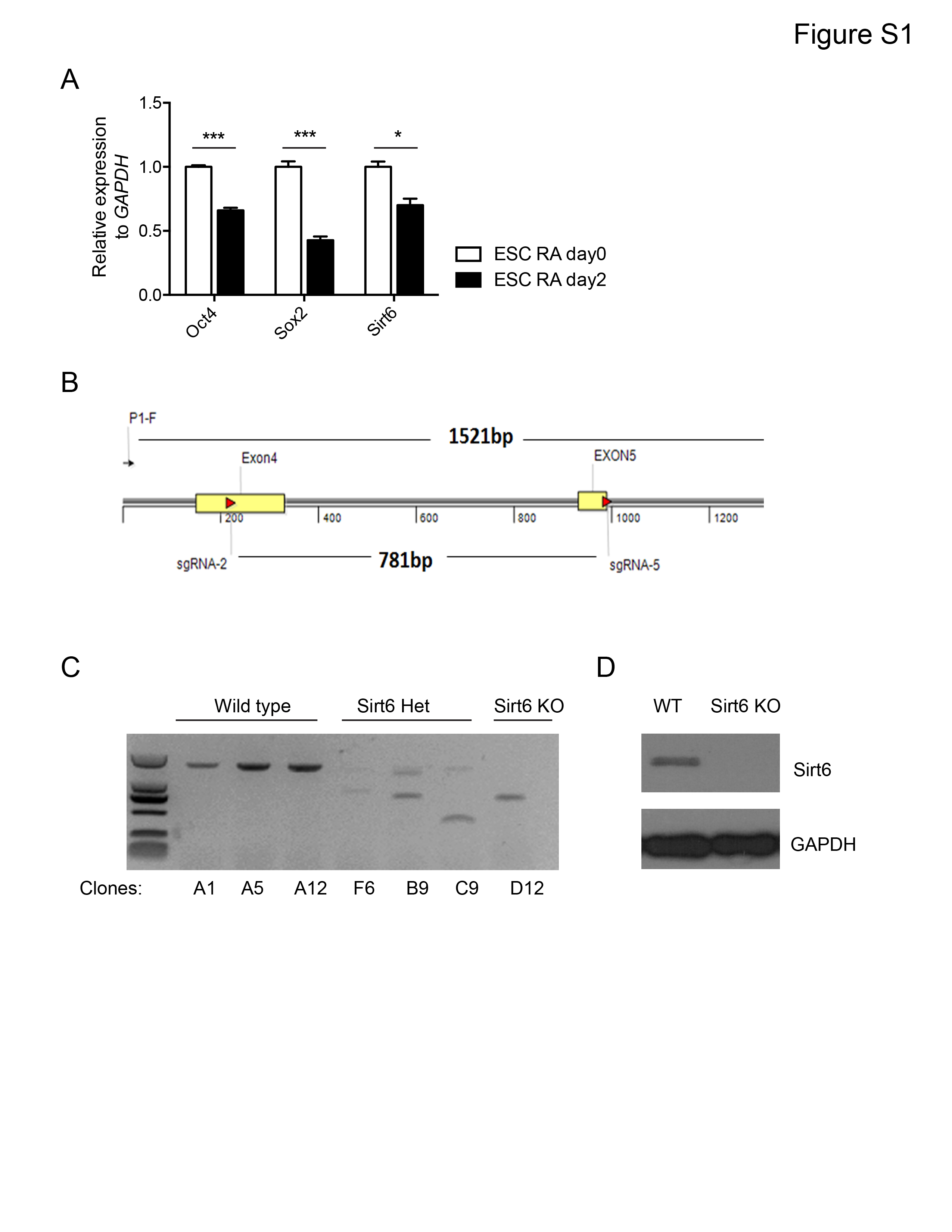

Supplement: Supplementary file 1 — Figure S1. (A): Sirt6 mRNA level after RA induced JM8A3 ES cells differentiation. All gene mRNA levels were measured by real time PCR; Oct4, Sox2 were represented as positive control, GAPDH was used as reference gene. All data were shown as mean values ± SD from three independent experiments. (*, p ≤ 0.05; **, p ≤ 0.01; ***, p ≤ 0.001). (B): Two CRISPR sgRNAs were designed to knockout region between exon4 and exon5 (highlighted in yellow) (781 bp); PCR primers and fragment used for genotyping was shown on the top. (C): Genotyping of the Sirt6 knockout JM8 ES clones by PCR; One pure knockout clone (D12) was selected. (D): Western blot to validate the expression of Sirt6 in Sirt6 knockout JM8 ES cells. (JPG 542 kb) [file 13287_2018_1109_MOESM1_ESM.jpg]

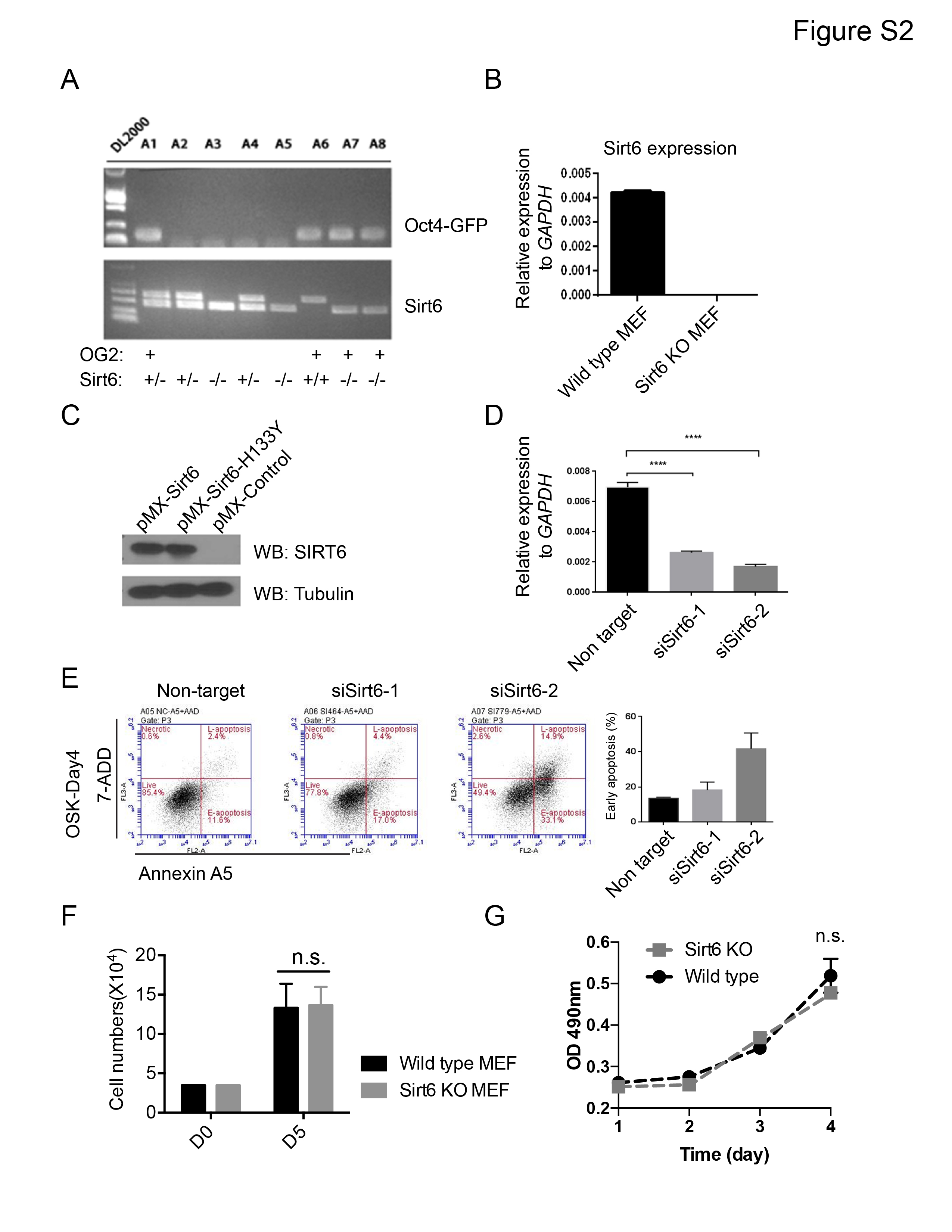

Supplement: Supplementary file 2 — Figure S2. (A): Sirt6/OG2 MEF genotype. Two sets of primers were used to genotype Sirt6 and Oct4-GFP allele separately. (B): Validation of Sirt6 mRNA by real-time PCR in Sirt6 wild-type and knockout MEFs. (C): Sirt6 expression measurement by Western blot after overexpression in Sirt6-null MEFs. (D): Validation of Sirt6 expression by real-time qPCR after two siRNA were transient transfection in mES-JM8 cells. (E): Cell apoptosis was measured in wild-type and Sirt6 null during reprogramming (F): Cell proliferation were measured in both wild-type and Sirt6-null MEFs after induced by OSKM at Day0 and Day5. (G): Viable cell numbers in both wild-type and Sirt6-null MEFs were assessed using a colorimetric assay with the light absorbance readout (OD 490 nm) at serial time points. (JPG 1001 kb) [file 13287_2018_1109_MOESM2_ESM.jpg]

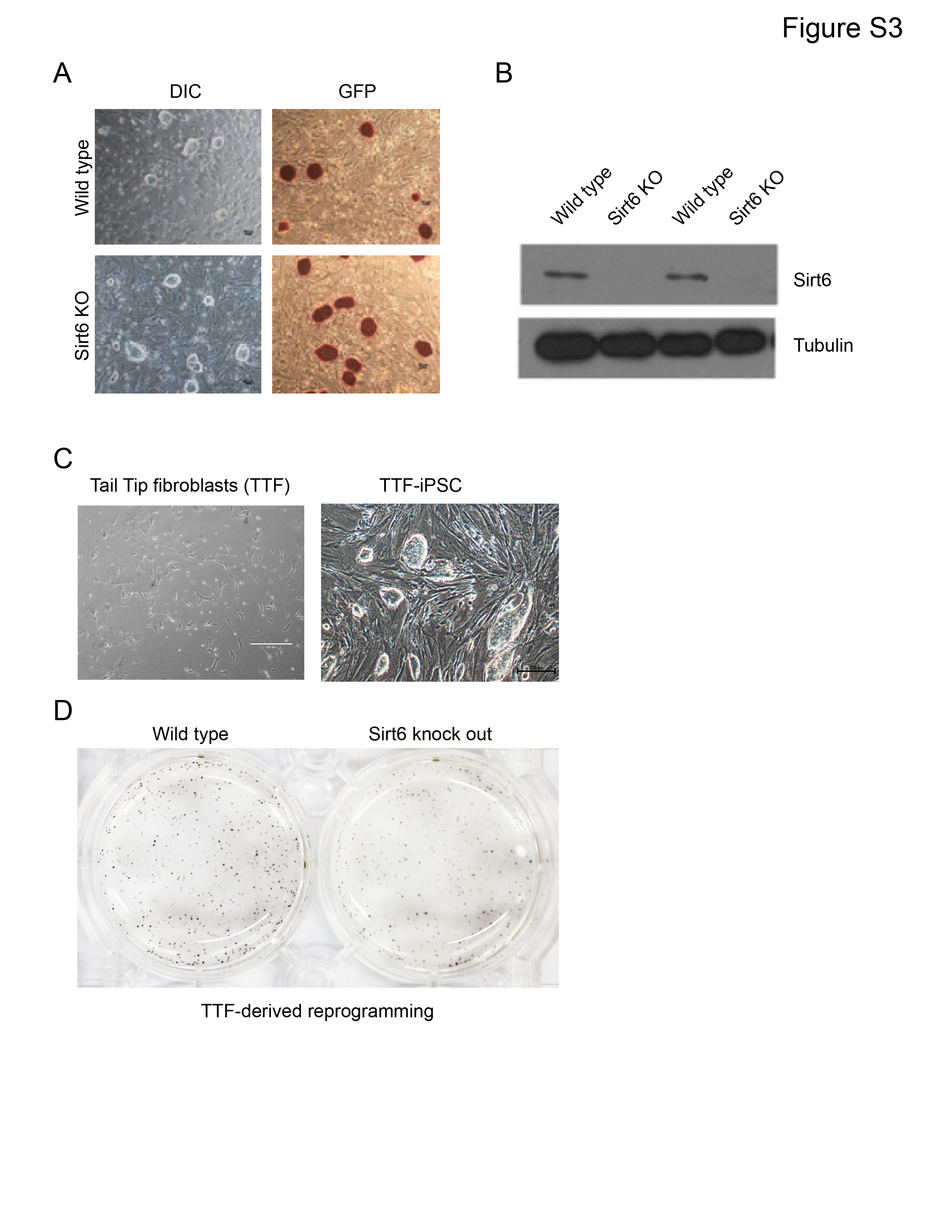

Supplement: Supplementary file 3 — Figure S3. (A): iPSC clones derived from wild-type and Sirt6-null Oct4-GFP MEF cells. IPS clones were picked and proliferated and identified by alkaline phosphatase staining. (B): Sirt6 expression was validated by Western blot in wild-type and Sirt6-null iPSC cells. (C): Tail-tip fibroblasts (TTF) and TTF-derived iPSCs. (D): Alkaline phosphatase staining showed reduced TTF reprogramming efficiency in Sirt6-null TTFs and wild-type TTFs. (JPG 2005 kb) [file 13287_2018_1109_MOESM3_ESM.jpg]

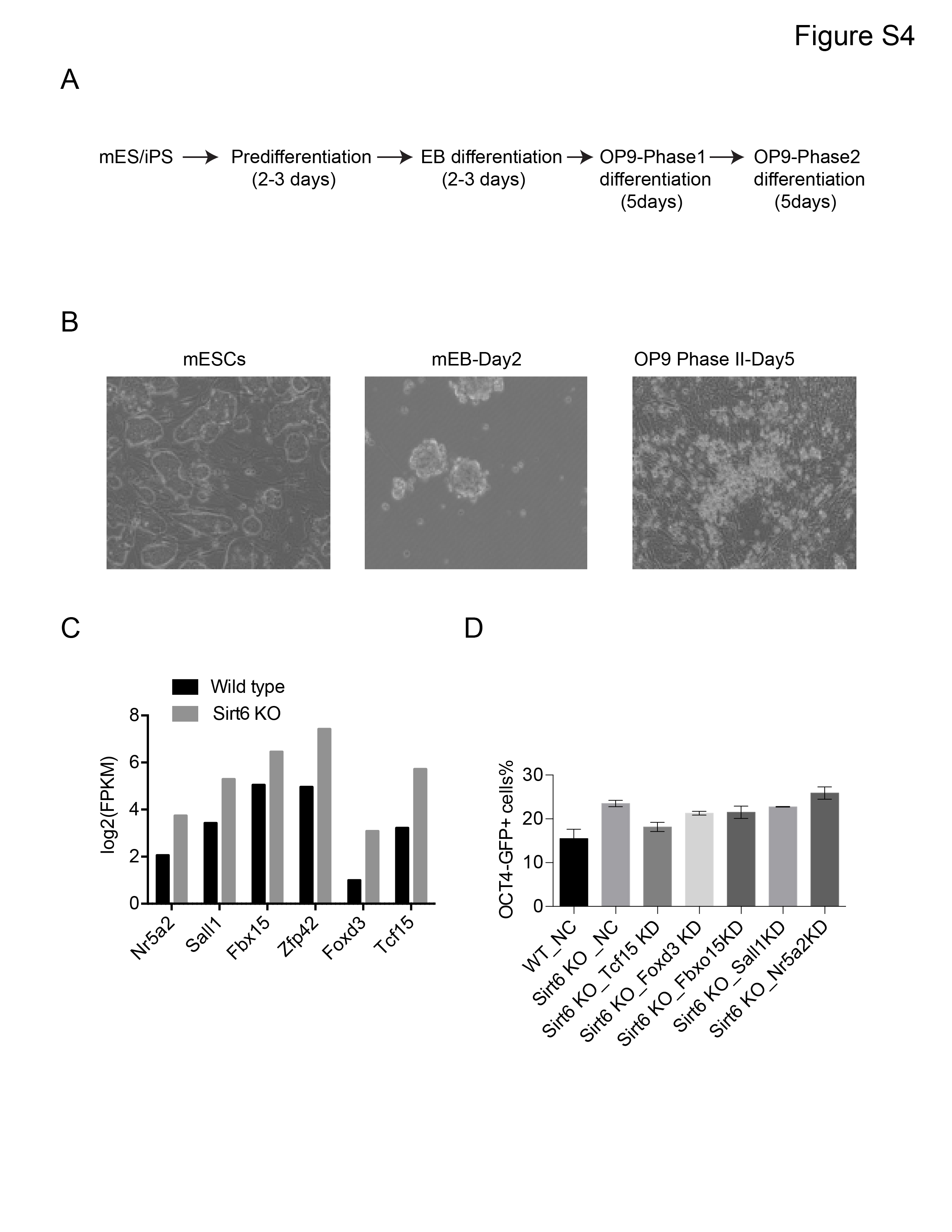

Supplement: Supplementary file 4 — Figure S4. (A): Three stages of in vitro hematopoietic differentiation from mES cells and iPS cells. Firstly, Mouse ES cell line or iPS-like cell line were cultured immediately in mESC pre-differentiation medium.; then these cells were digested into single cells by 0.05% Trypsin to generate EBs, the EBs were suspension cultured in EB differentiation medium. Finally, the EBs were digested by 0.05% Trypsin into single cells and be added to confluent OP9 cultures, and be co-cultured with OP9 cells for 6 days in differentiation medium. (B): Typical images of different stages of in vitro hematopoietic differentiation from mouse ES cells. (C): FPM showed the expression level of Nr5a2, Sall1, Sall3, Fbx15, Zfp42, Foxd3 and Tcf15 were upregulated in Sirt6-null iPS-like cell line. (D): Percentage of OCT4-GFP positive cells after 2 days of RA induced differentiation of Sirt6-null iPS-like cells transfected with Tcf15, Foxd3, Fbxo15, Sall1, and Nr5a2 siRNAs (Zfp42 KD) or negative control (NC) compared to wild-type iPSCs. Results of 3 replicates are summarized in the graph. (JPG 849 kb) [file 13287_2018_1109_MOESM4_ESM.jpg]
